# Cell Fitness: More Than Push-Ups

**DOI:** 10.3390/ijms22020518

**Published:** 2021-01-07

**Authors:** Adam James Ferrari, Ronny Drapkin, Rajan Gogna

**Affiliations:** 1 Penn Ovarian Cancer Research Center, Department of Obstetrics and Gynecology, University of Pennsylvania Perelman School of Medicine, Philadelphia, PA 19104, USA; adferr@pennmedicine.upenn.edu; 2 Graduate Program in Cell and Molecular Biology, University of Pennsylvania Perelman School of Medicine, Philadelphia, PA 19104, USA; 3 Department of Cancer Biology, University of Pennsylvania Perelman School of Medicine, Philadelphia, PA 19104, USA; 4 Basser Center for BRCA, Abramson Cancer Center, University of Pennsylvania School of Medicine, Philadelphia, PA 19104, USA; 5 Champalimaud Centre for the Unknown, 1400-038 Lisbon, Portugal

**Keywords:** cell competition, outcompete, cancer, cell fitness, epidermal stem cells, aging, oncogenic pathway, flower protein, hFWE, hippo pathway, YAP, TAZ, cell junction, COL17A1

## Abstract

Cell competition (CC) is a feature that allows tumor cells to outcompete and eliminate adjacent cells that are deemed less fit. Studies of CC, first described in *Drosophila melanogaster*, reveal a diversity of underlying mechanisms. In this review, we will discuss three recent studies that expand our understanding of the molecular features governing CC. In particular, we will focus on a molecular fitness fingerprint, oncogenic pathways, and the importance of cell junction stability. A fitness fingerprint, mediated by flower (hFWE) protein isoforms, dictates that cells expressing the flower-win isoforms will outcompete adjacent flower-loss-expressing cells. The impact of the flower protein isoforms is seen in cancer progression and may have diagnostic potential. The yes-associated protein (YAP) and TAZ transcription factors, central mediators of the oncogenic Hippo pathway, elevate peritumoral fitness thereby protecting against tumor progression and provide a suppressive barrier. Similarly, COL17A1 is a key component in hemidesmosome stability, and its expression in epidermal stem cells contributes to fitness competition and aging characteristics. The contributions of these pathways to disease development and progression will help define how CC is hijacked to favor cancer growth. Understanding these features will also help frame the diagnostic and therapeutic possibilities that may place CC in the crosshairs of cancer therapeutics.

## 1. Introduction

The ability of incipient cancer cells to overcome cellular and microenvironmental constraints depends on their ability to selectively rewire cell autonomous and cell non-autonomous attributes. These attributes, often referred to as the hallmarks of cancer, constitute the abilities acquired during multistep development of human tumors [1]. Recent studies suggest that in addition to the hallmarks already described, cell competition (CC) is a feature that allows tumor cells to outcompete and eliminate adjacent cells that are deemed less fit in comparison [2]. One simple example of CC is rapid cellular proliferation that results in the domination over more slower growing cells for physical space [3]. The molecular mechanisms that drive CC, by which unfit cells are culled, continue to emerge and include, for example, p53, Myc overexpression, nutrient competition, and mechanical pressure [2]. All of these play significant roles in development, tissue homeostasis, and aging, and are co-opted in cancer [2,3,4]. The field of CC is growing, and though we will only discuss three recent advances, the reader is encouraged to consider a more comprehensive review of the subject [5].

The first molecular component of CC was identified in *Drosophila melanogaster* by comparing the growth rates between cells carrying mutations in genes encoding ribosomal proteins (Minute+/−), and wild-type cells (Minute+/+) [6,7]. *Minute* wildtype cells outcompeted and eliminated *Minute* heterozygous cells in flies that contain both cell types. However, *Minute* mutant cells, in the absence of wildtype cells, remained viable, thereby uncovering a non-cell autonomous molecular mechanism contributing to CC [6]. In addition to the *Minute*-mediated CC in Drosophila, recent CC mechanisms have been identified in mammalian orthologs through a molecular fitness code, paradoxical oncogenic activation, and cell junction stability [8,9,10]. 

## 2. Cell Competition Can Be Mediated by a Molecular-Code Fitness Fingerprint

A recent study associated CC with a molecular fitness fingerprint of the flower (hFWE) protein [8], which was first discovered in *Drosophila* [11]. In human cells, flower is the transcriptional product of the C9ORF7 locus and is a transmembrane protein with several isoforms induced by alternative splicing [8]. Madan et al., 2019 determined that two of the four isoforms represented a decrease in fitness (loss), while the remaining two isoforms signified an increase in fitness (win), and the assortment of these isoforms created a fitness fingerprint for each cell.

Using a CRISPR-mediated hFWE knockout system in a breast cancer cell line, the authors were able to control the expression of individual hFWE isoforms and determine that each isoform alone did not alter cell proliferation or cell death. However, coculture of hFWE loss-expressing cells with win-expressing cells resulted in caspase-dependent apoptosis of the hFWE loss-expressing cells. This phenomenon was also determined to be cell-cell contact-dependent as conditioned media did not have a similar apoptotic effect [8].

Analysis of human tissues showed that all hFWE isoforms were expressed at low levels in normal tissue, but malignant and associated stroma exhibited a more diverse and spatially distinct pattern of hFWE expression. The primary tumor tissue was abundant for the win isoform, while the associated stroma expressed more of the loss isoform. Loss expression was found, in conjunction with elevated apoptotic genes, to be more prevalent in the layers of the stroma adjacent to the tumor tissue. Overall, both win and loss isoforms were poorly expressed in normal tissue, but this study showed that win was consistently expressed in tumor tissue and hFWE loss was more abundant in the stroma [8].

To address the functional impact of these observations, the authors showed in vivo that the knockdown of hFWE expression by RNA interference in tumor tissue reduced tumor volume and the probability of metastasis. The effect of knocking down hFWE in this system was completely rescued by ectopic hFWE Win expression. Conversely, over expression of the win isoform led to a significant increase in tumor growth [8].

To address the therapeutic potential of targeting hFWE, mice with hFWE knockout tumors were treated with mainstay therapies, including fluorouracil or cisplatin with docetaxel. Cancer cell lines with both high and low expression of win isoforms were subject to hFWE knockout and chemotherapy treatment in vivo. The combination resulted in a significant synergistic decrease in tumor volume and metastatic potential compared to each individual treatment alone [8].

This study identified a molecular code for a cell’s fitness fingerprint, showed its importance in cancer progression, and uncovered its potential therapeutic application. Interestingly, hFWE expression has also been linked to cancer incidence using models of skin papilloma [12]. Together, these studies support the notion that the hFWE fitness fingerprint could be developed into a diagnostic tool for cancer surveillance, perhaps by monitoring the assortment of hFWE isoforms in different tissues as a predictor of tumor development. Similarly, developing methods to activate the hFWE loss isoform in tumor tissue may pave the way for a novel therapeutic.

## 3. Oncogenic Pathways Can Drive Cell Competition

The Hippo pathway is a common regulator of cell proliferation, cell survival, and organ size that is stimulated by cellular contact, stress, and growth factors. It is consistently identified as an oncogenic driver and, similar to the flower protein, was first discovered in *Drosophila* [8,13]. Downstream effectors of the Hippo pathway are transcription factors yes-associated protein (YAP) and TAZ, which are commonly upregulated and have oncogenic functions in cancer. Hyperactivation of YAP and TAZ has been linked to organ overgrowth and cancer initiation [14]. However, Moya et al., 2019 recently identified tumor-suppressive properties of activated YAP and TAZ in peritumoral tissue.

Using a mouse model of induced intrahepatic cholangiocarcinoma (CCA) via Notch and Akt activation, the authors demonstrate that these tumors had elevated levels of YAP and TAZ with the expected upregulation of genes coding for cell proliferation, stress response, and wound healing. Hepatocytes of normal livers were barely detectable for YAP and TAZ. However, the hepatocytes surrounding the tumor tissue also exhibited YAP accumulation. Adeno-Associated Virus-Cre-mediated peritumoral ablation of YAP in this model showed accelerated tumor growth, suggesting that peritumoral YAP was acting to suppress further tumor progression. Furthermore, deletion of an endogenous YAP and TAZ negative-regulator, LATS1/2, in the surrounding tissue significantly reduced tumor burden, indicating that heightened YAP in the peritumoral tissue can restrain tumor growth. The simultaneous quadruple deletion of YAP, TAZ, and LATS1/2 in the peritumoral tissue increased cancer cell proliferation and tumor burden. Finally, activation of YAP and TAZ in the peritumoral tissue triggered non-apoptotic tumor cell death [9]. These data indicate that the levels of YAP and TAZ influence the fitness of the peritumoral tissue and impact the ability of the tumor cells to outcompete the surrounding cells for tissue real-estate.

As expected, ablation of tumoral YAP and TAZ greatly reduced tumor volume to a few residual tumors and almost completely eliminated the tumor. This confirms that YAP and TAZ are required for tumor maintenance when surrounded by WT hepatocytes. To analyze the tumor suppressive properties of YAP and TAZ in a metastatic tumor, the authors injected mice with melanoma-derived tumor cells that would home to the liver. Nearly 100% of the metastatic melanoma-based tumors were reduced upon peritumoral hepatocyte activation of YAP and TAZ. This showed that the CC mechanism of YAP and TAZ could be applied to both primary and migrant tumors [9].

Moya et al., 2019 showed that the Hippo pathway, specifically YAP and TAZ, elevate peritumoral fitness, thereby making these cells better suited to fend off the tumor cells. This paradoxical discovery identifies a robust tumorigenic pathway having space-specific tumor-suppressive properties. These results are consistent with other recent reports showing that YAP plays a major CC role in mouse development and in canine epithelial cells [15,16]. Hippo pathway inhibitors have been developed to treat cancers [17] but could pose as a tumor aid when targeting YAP and TAZ in the general tumor microenvironment. It would be interesting to see if we could promote YAP and TAZ activation solely in the peritumoral tissue as a therapy for both Hippo and non-Hippo mediated tumors. 

## 4. Cell Competition Dynamics Are Altered with Age

As we age, stem cells that once replenished our damaged skin become exhausted. This results in skin atrophy, fragility, impaired healing, and dyspigmentation [18,19]. An important factor in stem cell skin renewal is asymmetric cell division along the basement membrane. This process gives rise to two distinct daughter cells: one is a copy of the original stem cell and the other can differentiate into a non-stem cell fate along the membrane. Asymmetric cell division fills the void left by damaged keratinocytes [20]. COL17A1 is a transmembrane protein important in hemidesmosome linkage in epidermal stem cells, and its down regulation has been linked to skin fragility, atrophy, and a decreased epidermal stem cell population [21,22,23]. A recent study showed that aged tail skin from a mouse harbors fewer stem cells and that these stem cells undergo fewer cell divisions. Moreover, the hemidesmosomes were significantly decreased compared to younger tail skin. In addition, COL17A1 was significantly reduced and heterogeneous in its distribution, as described by whole mount immunostaining and immune-transmission electron microscopy. Administration of a protein synthesis inhibitor (cycloheximide) and a matrix metalloproteinase inhibitor (marimastat) showed that COL17A1 was the most unstable protein in hemidesmosomes, and its proteolysis was mediated by genomic stress, emphasizing its importance in skin homeostasis [10].

A genetically modified mouse model capable of tracing epidermal stem cells with different colors was used to show that the balance and number of stem cell clones was lost as the mouse aged. Moreover, the size of the dominant clones was correlated with high COL17A1 expression. These data suggest that COL17A1 may play a significant role in stem cell maintenance. Expanding on this concept further, the authors incorporated a COL17A1 knockout while tracing epidermal stem cells. Both heterozygous and homozygous COL17A1 knockout cells were significantly eliminated in the presence of wildtype cells and were eliminated from the epidermis. An in vitro 3D cocultures of keratinocytes having COL17A1 knockout plated at a 1:10 ratio with wildtype showed a significant elimination of COL17A1 knockout cells. These data showed that differential expression of COL17A1 contributes to CC [10].

The authors coupled the mechanism of COL17A1-mediated CC to the renewal properties of stem cells. It was observed that COL17A1 knockout cells divided asymmetrically perpendicular to the basement membrane, resulting in diminished renewal with concomitant epidermal thinning. Forced expression of COL17A1 rescued epidermal thinning, micro-delamination, and basal cell flattening. Impaired wound healing observed in the COL17A1 knockout mouse was also rescued by expression of COL17A1 in basal keratinocytes. Furthermore, compounds that could induce COL17A1 were identified and their administration increased colony number and size in a colony formation assay. Similar studies on skin morphogenesis revealed that cells with low Mycn are eliminated by CC either during early epidermal stratification or later in the differentiating epidermis [24]. Together, these data describe key components in epidermal stem CC during development, aging, and regeneration and show that COL17A1-mediatied competition could be manipulated therapeutically. Interestingly, COL17A1 has also been implicated in certain tumors [25,26,27], and it would be compelling to see if COL17A1 contributes to epithelial or fibrous connective tissue malignancies in the context of CC. Could targeting this transmembrane protein help reduce tumor growth or would it dislodge tumor cells promoting metastasis?

## 5. Summary

**Figure 1:**
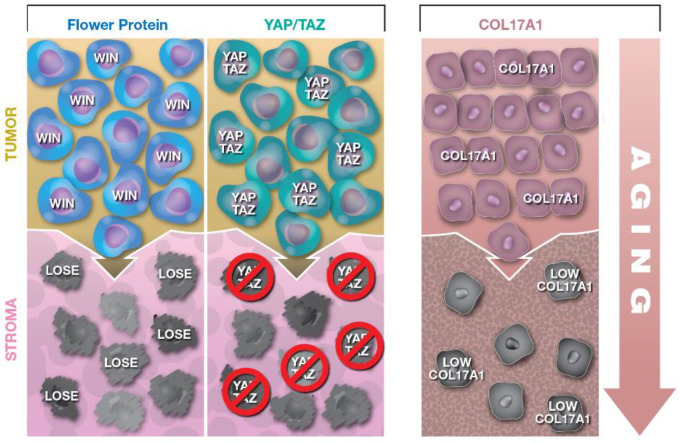
Illustration of cellular competition in cancer and aging skin. First column: hFWE win-expressing tumor cells outcompete adjacent stroma cells expressing hFWE loss. Second column: cells with a loss of YAP/TAZ expression in stroma cells are overtaken by YAP/TAZ abundant tumor cells. Third column: representation of epidermal stem cell competition in the context of COL17A1 expression. Low-COL17A1-expressing epidermal stem cells, which present characteristics of aged skin, are outcompeted by stem cells with higher COL17A1 expression.

CC is an important biological phenomenon and seems to be regulated by multiple mechanisms. A cohort of factors recently found to mediate CC include the hFWE protein isoforms [8], the YAP and TAZ transcription factors [9], and the COL17A1 transmembrane protein [10] (Figure 1). These key factors all contribute to a cell’s fitness and survival in the face of opposition. Other factors that contribute to CC will surely be identified, and defining their molecular contributions to CC will advance our understanding of diseases and expand therapeutic opportunities.
